# Supplementary material for: Correlation between Serum 25-Hydroxyvitamin D Level and Depression among Korean Women with Secondary Amenorrhea: A Cross-Sectional Observational Study
Source: Nutrients. 2022 Jul 10;14(14):2835. doi: 10.3390/nu14142835 (PMC9315875; doi:10.3390/nu14142835)
Supplement: Supplementary file 1 [file nutrients-14-02835-s001.zip › nutrients-1750384-supplementary.pdf]

Table S1. The relationship between depressive scores and biochemical variables by multivariate regression

variables	CES-D				K-HDRS			
	B	SE	β	*p*-value	B	SE	β	*p*-value
Age	-0.148	0.239	-0.620	0.539	0.125	0.100	0.199	0.219
BMI	0.461	0.406	0.167	0.262	-0.154	0.170	-0.126	0.370
25(OH)D(ng/mL)	-0.107	0.071	-0.247	0.140	-0.071	0.030	-0.375	0.021*
Estradiol (pg/ml)	0.028	0.032	0.168	0.376	0.007	0.013	0.098	0.586
Free testosterone (pg/ml)	-1.334	2.361	-0.095	0.575	-0.240	0.988	-0.039	0.809
Prolactin (ng/ml)	0.175	0.120	0.210	0.154	0.081	0.050	0.222	0.114
AMH (ng/ml)	-0.599	0.279	-0.375	0.038*	-0.313	0.117	-0.444	0.011*
LH (U/L)	-0.016	0.142	-0.024	0.913	-0.030	0.060	-0.102	0.623
FSH (U/L)	0.088	0.230	0.071	0.703	-0.072	0.096	-0.133	0.456
TSH (μmol/L)	0.088	1.423	-0.037	0.811	0.240	0.595	0.058	0.403
fT4 (ng/dl)	10.734	7.961	0.193	0.185	3.785	3.331	0.155	0.263
Total cholesterol (mg/dl)	0.020	0.056	0.056	0.693	0.004	0.022	0.028	0.204
Fasting glucose (mg/dl)	-0.102	-0.214	-0.214	0.160	0.007	0.030	0.033	0.234
	R^2 = 0.295<br>Adjusted R^2 = 0.066				R^2 = 0.363<br>Adjusted R^2 = 0.156			

*: p<0.05, **: p<0.005

Abbreviations: CES-D (Center for Epidemiological Studies-Depression Rating Scale), K-HDRS (Korean version of the Hamilton Depression Rating Scale), BMI (body mass index), 25(OH)D (25-hydroxyvitamin D), AMH (anti-Müllerian hormone), LH (luteinizing hormone), FSH (follicle stimulating hormone), TSH (thyroid stimulating hormone), fT4 (free thyroxine 4)
